# Identification of the evolutionarily conserved nuclear envelope proteins Lem2 and MicLem2 in *Tetrahymena thermophila*

**DOI:** 10.1016/j.gene.2019.100006

**Published:** 2019-01-22

**Authors:** Masaaki Iwamoto, Yasuhiro Fukuda, Hiroko Osakada, Chie Mori, Yasushi Hiraoka, Tokuko Haraguchi

**Affiliations:** a Advanced ICT Research Institute Kobe, National Institute of Information and Communications Technology, Kobe 651-2492, Japan; b Graduate School of Agricultural Science, Tohoku University, Osaki, 989-6711, Japan; c Graduate School of Frontier Biosciences, Osaka University, Suita 565-0871, Japan

**Keywords:** BAF, barrier-to-autointegration factor, DAPI, 4′,6‑diamidino‑2‑phenylindole, DDW, double distilled water, EDTA, ethylenediaminetetraacetic acid, ER, endoplasmic reticulum, GA, glutaraldehyde, HeH, helix-extension-helix, LAP2, lamina associated polypeptide 2, LEM, LAP2-Emerin-MAN1, MAC, macronucleus, MIC, micronucleus, MSC, Man1-Src1p-C-terminal, NE, nuclear envelope, NLS, nuclear localization signal, NPC, nuclear pore complex, ONM and INM, outer and inner nuclear membranes, PB, phosphate buffer, PBS, phosphate buffered saline, RRM, RNA recognition motif, TM, transmembrane, LEM domain, HeH domain, Man1-Src1p-C-terminal domain, MSC domain, Man1, Nuclear envelope, Nuclear dimorphism, Protist

## Abstract

Lem2 family proteins, i.e. the LAP2-Emerin-MAN1 (LEM) domain-containing nuclear envelope proteins, are well-conserved from yeasts to humans, both of which belong to the Opisthokonta supergroup. However, whether their homologs are present in other eukaryotic phylogenies remains unclear. In this study, we identified two Lem2 homolog proteins, which we named as Lem2 and MicLem2, in a ciliate *Tetrahymena thermophila* belonging to the SAR supergroup. Lem2 was localized to the nuclear envelope of the macronucleus (MAC) and micronucleus (MIC), while MicLem2 was exclusively localized to the nuclear envelope of the MIC. Immunoelectron microscopy revealed that Lem2 in *T. thermophila* was localized to both the inner and outer nuclear envelopes of the MAC and MIC, while MicLem2 was mostly localized to the nuclear pores of the MIC. Molecular domain analysis using GFP-fused protein showed that the N-terminal and luminal domains, including the transmembrane segments, are responsible for nuclear envelope localization. During sexual reproduction, enrichment of Lem2 occurred in the nuclear envelopes of the MAC and MIC to be degraded, while MicLem2 was enriched in the nuclear envelope of the MIC that escaped degradation. These findings suggest the unique characteristics of *Tetrahymena* Lem2 proteins. Our findings provide insight into the evolutionary divergence of nuclear envelope proteins.

## Highlights

• Conserved nuclear envelope proteins Lem2 and MicLem2 are identified in Tetrahymena.
• Lem2 is localized to the nuclear envelope of the macronucleus and the micronucleus.
• MicLem2 is localized to the nuclear pore complex of the micronucleus.
• In sexual reproduction, Lem2 is enriched to the nuclei assigned to degradation.
• MicLem2 is enriched to the micronuclei that are escaped from degradation.

## Graphical abstract

**Figure:**
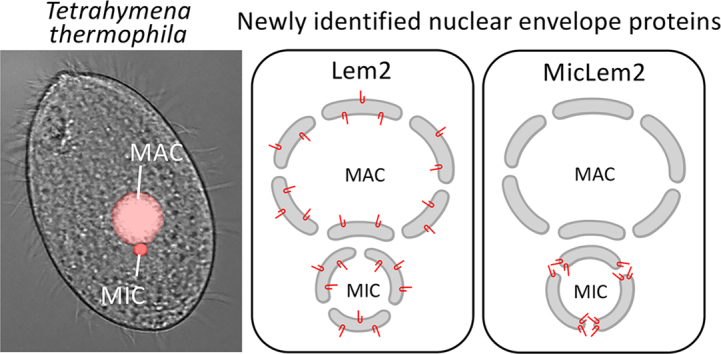

## 1 Introduction

The nuclear envelope (NE) is a cell structure that physically and functionally separates genomic DNA from the cytoplasm. The NE is composed of the outer and inner nuclear membranes (ONM and INM, respectively), which are connected to the pore membrane (reviewed in Goldberg and Allen, 1995; De Magistris and Antonin, 2018). In addition to this conserved structure, the nuclear lamina, a protein meshwork composed of lamins (type V intermediate filament proteins), is underneath the INM only in metazoan (reviewed in de Leeuw et al., 2018). The nuclear lamina does not exist in organisms other than metazoa including yeasts, plants, and protozoa (reviewed in Cohen et al., 2001; Iwamoto et al., 2016). The INM contains INM-specific integral membrane proteins; the ONM continues into the endoplasmic reticulum (ER) and thus contains many ER proteins. To date, several hundred putative transmembrane proteins have been identified in mammalian cells through proteomics analysis (Schirmer et al., 2003; Korfali et al., 2012; de Las Heras et al., 2013).

The LEM-domain proteins are among the best characterized NE proteins. LAP2, emerin, and MAN1 are the founding members of the LEM domain NE proteins, which also bind A-type lamins (Lee and Wilson, 2004). Members of the LEM-domain proteins contain a common bi-helical motif, known as the LAP2-Emerin-MAN1 (LEM) domain, in their N-termini (Dechat et al., 2000; Brachner and Foisner, 2011). The LEM domain, composed of approximately 40 amino acid residues (pfam03020), binds to barrier-to-autointegration factor (BAF), a DNA-binding protein; its binding to the LEM domain connects the NE to chromatin (Shumaker et al., 2001; Haraguchi et al., 2001; Haraguchi et al., 2008). The LEM-domain proteins contribute to genome organization and nuclear integrity by binding to BAF and A-type lamins in metazoan cells (Margalit et al., 2005; Wagner and Krohne, 2007; Pałka et al., 2018).

**Fig. 1:**
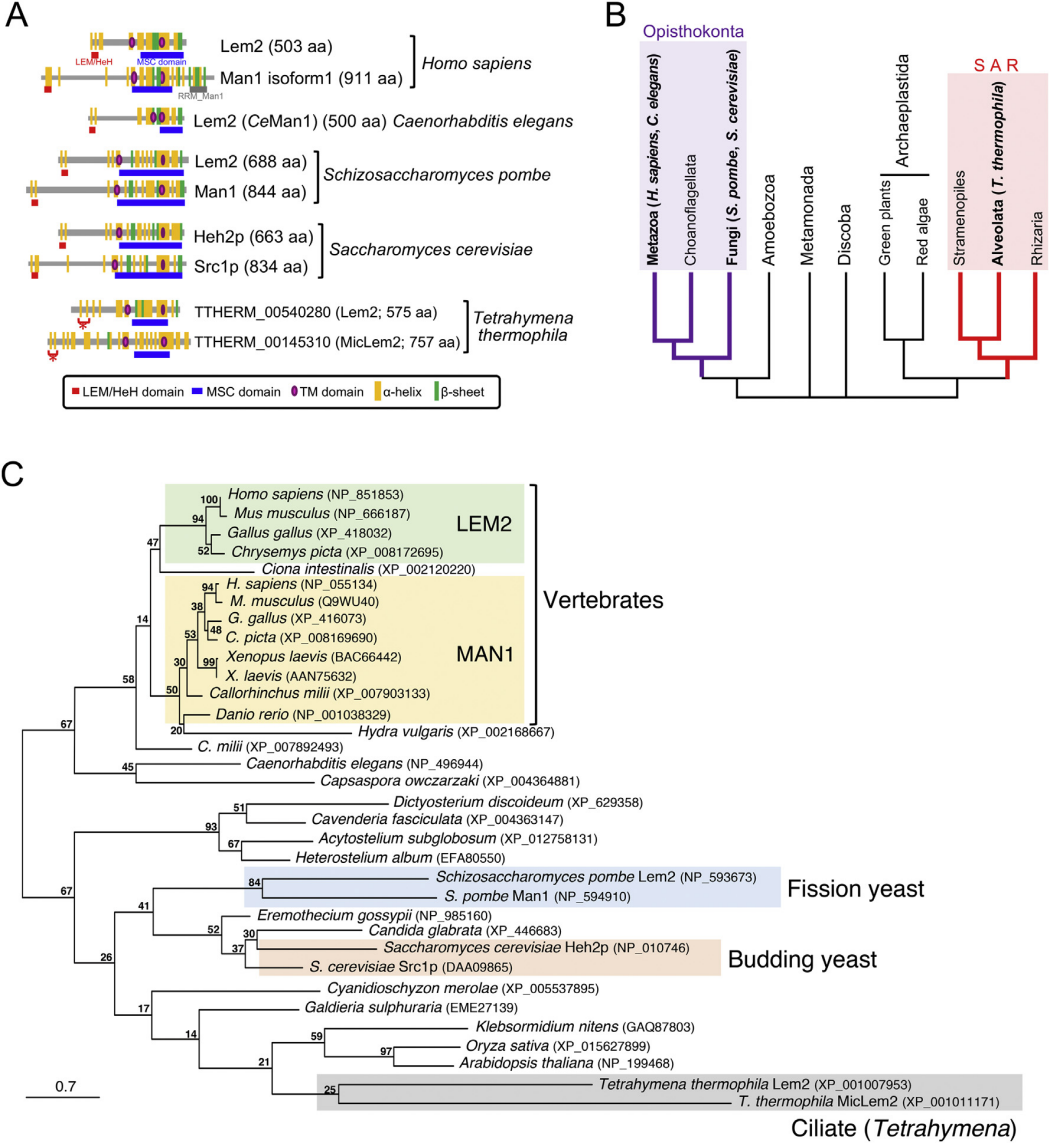
Lem2/Man1 proteins of *Tetrahymena thermophila* and other eukaryotes. A. Distributions of conserved domains and predicted secondary structures within the Lem2/Man1 proteins. The red and blue underlines indicate LEM/HeH and MSC domains, respectively. The gray underline indicates the RRM_Man1 domain. The purple ellipses represent the positions of transmembrane (TM) segments. The orange and green boxes represent the predicted α-helices and β-sheets, respectively. The asterisks indicate two N-terminal α-helices of *T. thermophila* Lem2 homologs that appear to be structurally related to the LEM/HeH domain. B. Latest evolutionary tree of eukaryotes, modified from the paper of Adl et al., 2018. C. Phylogenetic tree of Lem2/Man1 family proteins. The tree was reconstructed using the maximum likelihood method. The bootstrap values evaluated by 1000 replications are indicated on each node. The scale bar represents the number of expected amino acid residue substitutions per site. (For interpretation of the references to color in this figure legend, the reader is referred to the web version of this article.)

Among LEM-domain proteins, Lem2 family proteins are widely conserved from yeasts to humans, whereas other LEM-domain proteins such as LAP2 and emerin are metazoan-specific (Lee et al., 2000; Mans et al., 2004; Brachner and Foisner, 2011). The Lem2 family proteins share the LEM domain at the N-terminus and Man1-Src1p-C-terminal (MSC) domain (pfam09402) at the C-terminus, in addition to the two transmembrane helices in the middle region (Fig. 1A). Two paralogous proteins, Lem2 and Man1, are present in mammalian cells (Brachner et al., 2005) and only one homolog, Lem2, exists in *Caenorhabditis elegans* (Barkan et al., 2012). In these organisms, the Lem2 family proteins share the canonical LEM domain at the N-terminus. However, two paralogous proteins in fungi – Heh2p and Heh1p/Src1p in *Saccharomyces cerevisiae* (King et al., 2006), and Lem2 and Man1 in *Schizosaccharomyces pombe* (Hiraoka et al., 2011; Gonzalez et al., 2012) – do not share the canonical LEM-domain but instead share the non-canonical LEM-related helix-extension-helix (HeH) domain (pfam12949) at the N-terminus. Although NE proteins have been extensively studied in organisms belonging to the Opisthokonta supergroup such as yeasts and mammals, whether their homologs are present in other eukaryotic phylogenies remains unclear. NE proteins have not been experimentally identified in eukaryotes other than those from the Opisthokonta supergroup, except for *Dictyostelium* in the Amoebozoa supergroup (Batsios et al., 2016).

*Tetrahymena*, a ciliated unicellular organism, is a unique model eukaryote because it contains two functionally and structurally distinct nuclei, the macronucleus (MAC) and micronucleus (MIC), within each cell. The MAC is somatic and performs functions such as gene expression throughout all life cycle stages, whereas the MIC is a germline nucleus that generates differentiated macronuclei and micronuclei during sexual reproduction (Orias et al., 2011; Karrer, 2012). It has been reported that the nuclear pore complexes (NPCs) in the MAC and MIC of *Tetrahymena thermophila* are composed of partly different nucleoporins (Iwamoto et al., 2009; Iwamoto et al., 2017; Iwamoto et al., 2018), some of which dynamically change their localization in the NE of the developing nuclei during nuclear differentiation (Iwamoto et al., 2015). In this process, redundant NE structures, which have two sets of the double-membrane NE structure, form on the NE of the MIC-derived developing nuclei, are selected for further development (Iwamoto et al., 2015; Yang et al., 2017). Meanwhile, MIC-derived nuclei that are not selected for development are degraded. Thus, these nuclei must be recognized for degradation, which likely occurs through the NE structures. Thus, studies of the NE structure are important for understanding the process of nuclear differentiation in *Tetrahymena*. However, the NE proteins either on the MAC or MIC have never been identified. Additionally, lamins are not encoded by the genome, and thus, lamin-dependent laminar structures do not form in either the MAC or MIC underneath the INM in *Tetrahymena* (Iwamoto et al., 2016).

In this study, we detected Lem2 family NE proteins in *T. thermophila* based on localization analysis involving fluorescence microscopy and immunoelectron microscopy; *T. thermophila* is in the Alveolata group belonging to the SAR supergroup, distant from the Opisthokonta supergroup. Their dynamic changes in NE localization during nuclear differentiation were also analyzed to determine the functions of these evolutionarily conserved NE proteins.

## 2 Materials and methods

### 2.1 Tetrahymena strains, culture conditions, and induction of conjugation

Inbred strains CU427 [*chx1-1*/*chx1-1* (CHX1; cy-s, VI)] and CU428 [*mpr1-1*/*mpr1-1* (MPR1; mp-s, VII)] were used as wild-type cells for control experiments. They were also used as parental strains to generate cell lines ectopically expressing GFP-fused proteins (GFP-Lem2 and GFP-MicLem2). Cells were grown in shallow culture medium composed of 1.5% proteose-peptone (Difco, Detroit, MI), 0.5% yeast extract (Difco), 0.5% d-glucose, and 20 μM FeCl_3_, without agitation or aeration. To induce conjugation, strains of two different mating types in the mid-logarithmic phase of growth were separately washed with starvation medium (10 mM Tris-HCl, 40 nM CaCl_2_, pH 7.5) by low-speed centrifugation at 700*g* for 1 min, and then resuspended in starvation medium at a cell density of ~1 × 10^5^ cells/mL. After incubation for ~18 h, the two starved strains were mixed to induce conjugation. The cells were maintained at 30 °C for culture and conjugation.

### 2.2 Domain searching, secondary structure prediction, and phylogenetic analysis

To predict the secondary structure, the candidate proteins deduced from these two genes were analyzed using PSIPRED (http://bioinf.cs.ucl.ac.uk/psipred/) to identify the structural motifs of α-helices and β-sheets and TMHMM Server v. 2.0 software (http://www.cbs.dtu.dk/services/TMHMM-2.0/) to identify transmembrane domains.

Potential orthologous sequences of Lem2 and Man1 were collected from the NCBI database by searching the C-terminal MSC domain (pfam09402). The collected sequences were aligned using the multiple sequence alignment program MAFFT v7.294b (Katoh et al., 2002) with the globalpair and maxiterate options (Supplemental data 1; Supplemental Fig. S1). All gap regions were eliminated, and the remaining 112 amino acid residues (Supplemental data 2) showing the best alignment at the amino acid level was assigned on the conserved C-terminal MSC domain (Supplemental Fig. S1B); this sequence was utilized for phylogenetic tree reconstruction. For maximum likelihood (ML) phylogenetic analysis, the best substitution model and optional parameters were evaluated using Aminosan (Tanabe, 2011), and LG + I + G + F was suggested as the best setting. The ML phylogenetic relationships were calculated using raxmlGUI (Ver. 1.31) (Silvestro and Michalak, 2012), and 1000 replicated trees were reconstructed from the same model to evaluate the thorough bootstrap value.

### 2.3 Plasmid construction and transformation

To clone the cDNAs of *TTHERM_00540280* and *TTHERM_00145310*, the total RNA fraction was isolated from vegetatively growing CU427 cells using TRIzol reagent (Thermo Fisher Scientific, Waltham, MA). This fraction was used as a template to synthesize first-strand cDNAs of these genes by RT-PCR using SuperScript III (Thermo Fisher Scientific) with the oligo(dT) primers, and then amplified by PCR using PrimeSTAR reagent (Takara, Otsu, Japan) and specific primers (Supplemental Table S1). To generate an ectopic expression vector of proteins fused with GFP at their N-termini, the PCR products were treated with *Xho*I and *Apa*I and inserted into the multicloning site of the ribosomal DNA-based plasmid vector pIGF1 (Malone et al., 2005). To generate an ectopic expression vector of proteins fused with GFP at their C-termini, the PCR products were treated with *Xho*I and *Kpn*I and inserted into the pIGF1C vector (Iwamoto et al., 2017).

The plasmids carrying the transgenes were introduced at 10 h after the induction of conjugation into mating-paired cells by electroporation using Gene Pulser (BioRad, Hercules, CA) under previously described pulse conditions (Iwamoto et al., 2014). After electroporation, the cells were suspended in culture medium and aliquoted into 96-well plates. After overnight incubation, paromomycin sulfate (Duchefa Biochemie, Haarlem, Netherlands) was added to each well as a selection drug at a final concentration of 120 μg/mL. The cells exhibiting resistance to paromomycin were selected using paromomycin sulfate up to a concentration of 500 μg/mL and maintained in the culture medium containing the same concentration of the drug.

### 2.4 Expression of GFP-tagged proteins

To observe GFP-Lem2, CdCl_2_ was added at a final concentration of 0.01 μg/mL to growing cells or 0.001 μg/mL to conjugating cells. To observe Lem2-GFP, CdCl_2_ was added at a final concentration of 0.1 μg/mL. To observe GFP-MicLem2, the cells were cultured in medium without CdCl_2_ because leaky expression was sufficient to observe GFP fluorescence.

### 2.5 Fluorescence microscopy for fixed cells

*Tetrahymena thermophila* cells expressing GFP-tagged proteins were collected by low-speed centrifugation and fixed with cold methanol for 30 min at −30 °C, and then further fixed with 4% formaldehyde for 30 min at room temperature (~25 °C). After washing three times with phosphate-buffered saline (PBS) for 10 min each, the fixed cells were counterstained with 0.05 μg/mL 4′,6‑diamidino‑2‑phenylindole (DAPI) and mounted between coverslips with 25% (v/v) glycerol in PBS. Fluorescence images were obtained using a fluorescence microscope IX-70 (Olympus, Tokyo, Japan) with an oil-immersion objective lens UApo 40×/1.35 oil or PlanApo N60×/1.40 oil (both from Olympus) equipped in the DeltaVision microscope system (GE Healthcare, Little Chalfont, UK). Twenty z-stack images at 0.5-μm intervals were acquired for each cell and deconvolved using softWoRx software (GE healthcare).

### 2.6 Immuno-electron microscopy

Vegetative growing cells expressing GFP-Lem2 or GFP-MicLem2 were fixed for 5 min with formaldehyde at a final concentration of 4% by adding 16% stock solution (Polysciences, Warrington, PA) to the culture medium. The cells were collected by low-speed centrifugation at ~700 *g* and resuspended in 4% formaldehyde diluted in 0.1 M phosphate buffer (PB; pH 7.5). Next, the cells were incubated for 25 min at room temperature for fixation. The fixed cells were washed three times with PB for 10 min each (hereafter, the same washing procedure was performed for all treatments), and permeabilized with 0.1% saponin (Nacalai Tesque, Inc., Kyoto, Japan) diluted in PB for 15 min. To activate the antigens, the fixed cells were treated with 0.01% trypsin and 0.1 mM EDTA in PB for 30 min at 25 °C. After blocking with 1% bovine serum albumin for 1 h, the fixed cells were treated with 5 μg/mL anti-GFP rabbit polyclonal antibodies (Rockland, Limerick, PA) overnight at 4 °C and then with 0.2 μg/mL anti-rabbit IgG goat poly-Fab' labelled with both Alexa Fluor 594 and 1.4-nm Nanogold particles (Nanoprobes, Yaphank, NY) for 2 h.

Post immuno-labelling fixation was done with 2.5% glutaraldehyde (Nacalai Tesque, Inc.) for 30 min at room temperature, followed by three washes with 100 mM lysine in PB and one wash with PB for 10 min each. After an additional three washes with 50 mM Hepes buffer (pH 5.8) and one wash with double distilled water (DDW) for 3 min each, the immuno-stained cells were treated with silver-enhancement reagent (Tange et al., 2016) for 3 min at 25 °C. The reaction was abated by washing three times with DDW for 5 min each. The cells were embedded in a thin layer of 0.5% low-melting point agarose (#50101, Lonza, Basel, Switzerland) on a glass-bottomed dish, and post-fixed with 1% osmium tetroxide for 15 min. After washing three times with DDW, the cells were stained with 2% uranyl acetate for 1 h. The reaction was stopped by washing three times with DDW. The sample was dehydrated with sequentially increasing concentrations of ethanol (from 30 to 100%). Next, the sample was substituted with epoxy resin by sequentially increasing the concentrations of Epon812: 10%, 30%, 50%, 70%, and 90% in ethanol for 20 min each, and 100% three times for 3 h each. The resin was polymerized at 60 °C for 48 h. The resin block was sliced into ultrathin sections using an ultramicrotome (EM UC6, Leica Microsystems, Wetzlar, Germany). After staining with 4% uranyl acetate for 15 min and lead citrate (Sigma-Aldrich, St. Louis, MO) for 1 min, the sections were observed with a JEM-1400 transmission electron microscope (JEOL, Tokyo, Japan) at an acceleration voltage of 80 kV.

All procedures were performed at room temperature (~25 °C) unless otherwise stated.

## 3 Results

### 3.1 Identification of two Lem2 family proteins, Lem2 and MicLem2, in Tetrahymena

Lem2 family proteins have three conserved domains, an N-terminal LEM/HeH domain (hereafter, LEM domain), C-terminal Man1-Src1p-C-terminal (MSC) domain, and two transmembrane helices in the middle region (Fig. 1A). To identify its homologous proteins in the ciliate *T. thermophila*, which belongs to the Alveolata group (Fig. 1B), we searched for proteins possessing the MSC domain in the NCBI protein database (https://www.ncbi.nlm.nih.gov/protein/); the MSC domain sequences of human Lem2, human Man1, *C. elegans* Lem2, *S. pombe* Lem2, *S. pombe* Man1, *S. cerevisiae* Heh2p, and *S. cerevisiae* Src1p were used as a query sequence in BLAST. The database listed numerous proteins (~50) in *T. thermophila*. We selected the proteins with a molecular size of 500–1000 amino acids from the list and then further evaluated the presence of the transmembrane helix in the selected proteins. Only one protein (TTHERM_00145310, 757 amino acids (aa)) with two transmembrane helices and the expected molecular size was found when *S. pombe* Lem2, *S. pombe* Man1, and *S. cerevisiae* Src1p were used as query sequences, whereas no proteins were found when human Lem2, human Man1, and *C. elegans* Lem2 were used. We next searched for homologous proteins in *T. thermophila* by entering an entire amino acid sequence of TTHERM_00145310 as a query in BLAST and found one homolog (TTHERM_00540280, 575 aa) with two transmembrane helices. Both TTHERM_00145310 and TTHERM_00540280 possess one MSC domain in their C-termini and two transmembrane helices in the middle region (Fig. 1A). Although no canonical LEM domain was found based on the similarity of amino acid sequences in these proteins, they contain two α-helices in their N-termini (indicated by red asterisks in Fig. 1A; also see Supplemental Fig. S1A). These molecular features resembled those of Lem2/Man1-related proteins found in organisms from the Opisthokonta supergroup, which is distant from *Tetrahymena* in the SAR supergroup (Fig. 1B). Thus, we further characterized these proteins as candidate Lem2 proteins in *T. thermophila*.

In addition to these two Lem2-related proteins, we also found one additional candidate protein (TTHERM_00382430, 165 aa) by searching the MSC domain in the NCBI database as a query. However, compared to the other two candidates, this protein was too small because it lacked most of the N-terminal domain, the distance between the two transmembrane helices was small, and its expression in cells was very low according to the gene expression profile of the *Tetrahymena* genome database (http://ciliate.org/index.php/home/welcome). Therefore, we did not include this protein as a Lem2-related protein.

To understand the evolutionary relationship of TTHERM_00145310 and TTHERM_00540280 to other Lem2 family proteins in various organisms, we performed phylogenetical analysis. We first searched consensus sequences among all Lem2-related proteins listed in Fig. 1C; aligned amino acid sequence for full length of those proteins are shown in Supplemental data 1. The resulting conserved region was 112 amino acid residues in the MSC domain (sequence information of the conserved regions is shown in Supplemental data 2). These 112 amino acid residues were used to evaluate the phylogenetical relationship and bootstrap values. The molecular phylogenetic tree showed that vertebrate Man1 diverges from Lem2 at the branch point to the vertebrates, and thus the two candidate proteins in *T. thermophila* diverged independently from the that of vertebrate Lem2 and Man1 (Fig. 1C). Because both candidate proteins lacked the C-terminal domain characteristic to Man1 (Fig. 1A), both proteins were considered as Lem2-related proteins. These Lem2-related protein homologs in *T. thermophila* were conserved in the genus *Tetrahymena*, but not found in the genus *Paramecium*.

### 3.2 TTHERM_00540280 is localized to the NEs of both the MAC and MIC, and TTHERM_00145310 is localized only to the NEs of the MIC

**Fig. 2:**
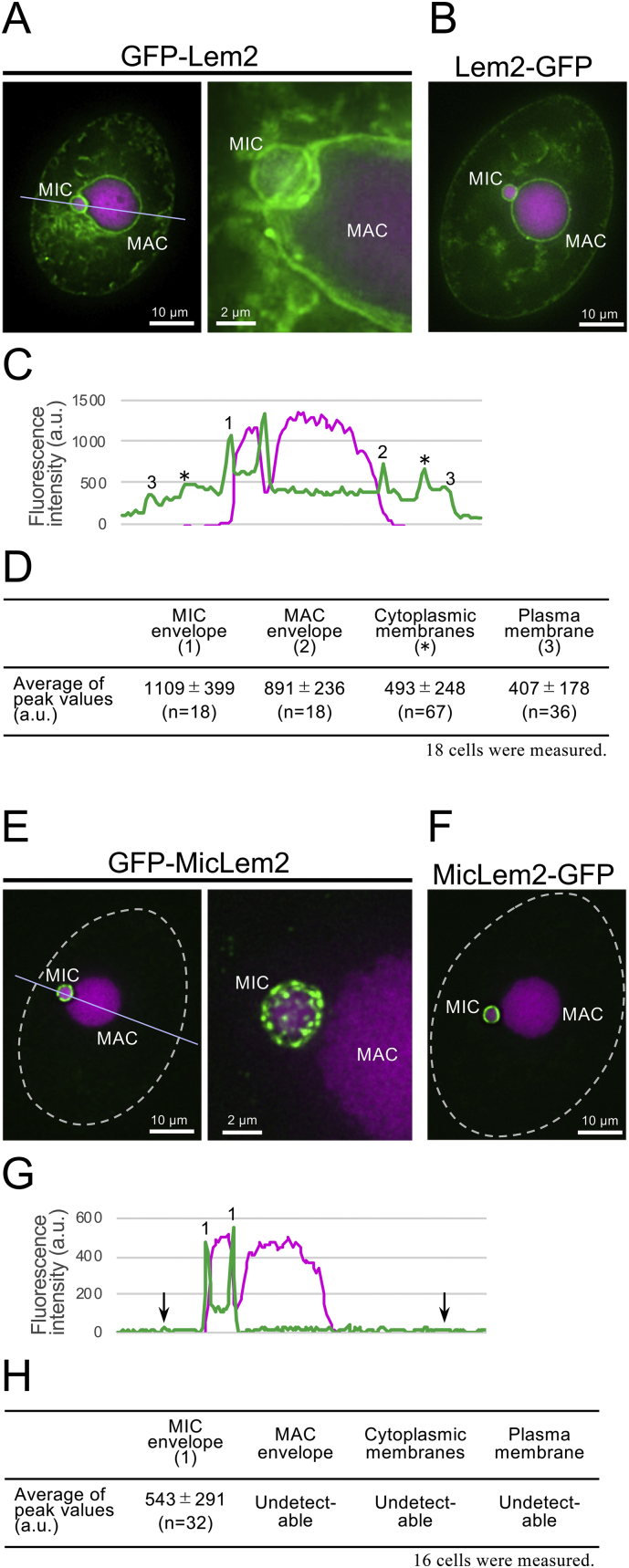
Subcellular localization of GFP-tagged Lem2 proteins in *T. thermophila*. A typical image is shown. Cells expressing GFP-tagged proteins were fixed and counterstained with DAPI. “MIC” and “MAC” indicate micro- and macronucleus, respectively. A. Localization of GFP-Lem2. The left panel shows a single focal plane image of the deconvoluted 3D-images (see Materials and methods). The right panel is a magnified view of the projected images of 5 focal planes. B. Subcellular localization of Lem2-GFP. C. Quantification of fluorescence intensity of GFP along a line indicated in (A). Green and red lines indicate levels of GFP and DAPI fluorescence, respectively. The marks of (1), (2), (⁎), and (3) indicate the positions of MIC NE, MAC NE, cytoplasmic membranes, and plasma membrane, respectively. D. Average values of fluorescence intensity in the corresponding positions shown in (C). E. Subcellular localization of GFP-MicLem2. The left panel shows a single focal plane image of the deconvoluted 3D-images. The right panel is magnified view of the projected images of 5 focal planes. F. Subcellular localization of MicLem2-GFP. G. Quantification of fluorescence intensity of GFP along a line indicated in (E). Green and red lines indicate levels of GFP and DAPI fluorescence, respectively. The marks of (1) and arrows indicate the positions of MIC NE and plasma membrane, respectively. H. Average values of fluorescence intensity in the corresponding positions shown in (G). The white broken line represents the outline of the cell. The scale bars are indicated in each panel. (For interpretation of the references to color in this figure legend, the reader is referred to the web version of this article.)

Lem2 proteins in other organisms are localized in the NE. To characterize Lem2 candidate proteins in *Tetrahymena*, we examined the subcellular localization of these proteins by observing GFP-tagged proteins expressed in vegetative *T. thermophila* cells. Fluorescence signals of the TTHERM_00540280 protein fused to GFP at its N-terminus (GFP-Lem2) were detected mostly in the NE of both the MAC and MIC and some minor signals were detected in the cytoplasmic and plasma membranes (Fig. 2A). Quantification of the fluorescence intensities along a line crossing through the cells (while line in Fig. 2A) support this finding (Fig. 2C, D). TTHERM_00540280 proteins fused to GFP at the C-terminus (Lem2-GFP) showed the same localization (Fig. 2B), suggesting that TTHERM_00540280 is an NE protein. These results demonstrate that the molecular features of this protein in *Tetrahymena* are similar to those of LEM2 in other organisms, and thus we named this protein as Lem2. In contrast, the fluorescence signals of the TTHERM_00145310 protein fused to GFP at the N-terminus (GFP-MicLem2) or C-terminus (MicLem2-GFP) were detected only in the NE of the MIC in both cases (Fig. 2E, F), and thus we named this protein as MicLem2. Quantification of the fluorescence intensity along a line crossing through the cells (white line in Fig. 2E) supported that this protein was predominantly localized in the MIC NE (Fig. 2G, H). Interestingly, GFP-MicLem2 exhibited punctate localization on the NE of the MIC (see right panel of Fig. 2E), while GFP-Lem2 showed uniform localization on the NE of the MAC and MIC, as observed for known Lem2 family proteins from other organisms (Fig. 2A) (Brachner et al., 2005; Hiraoka et al., 2011; Gonzalez et al., 2012; Barkan et al., 2012). Thus, Lem2 and MicLem2 localize at different structural domains within the NE.

**Fig. 3:**
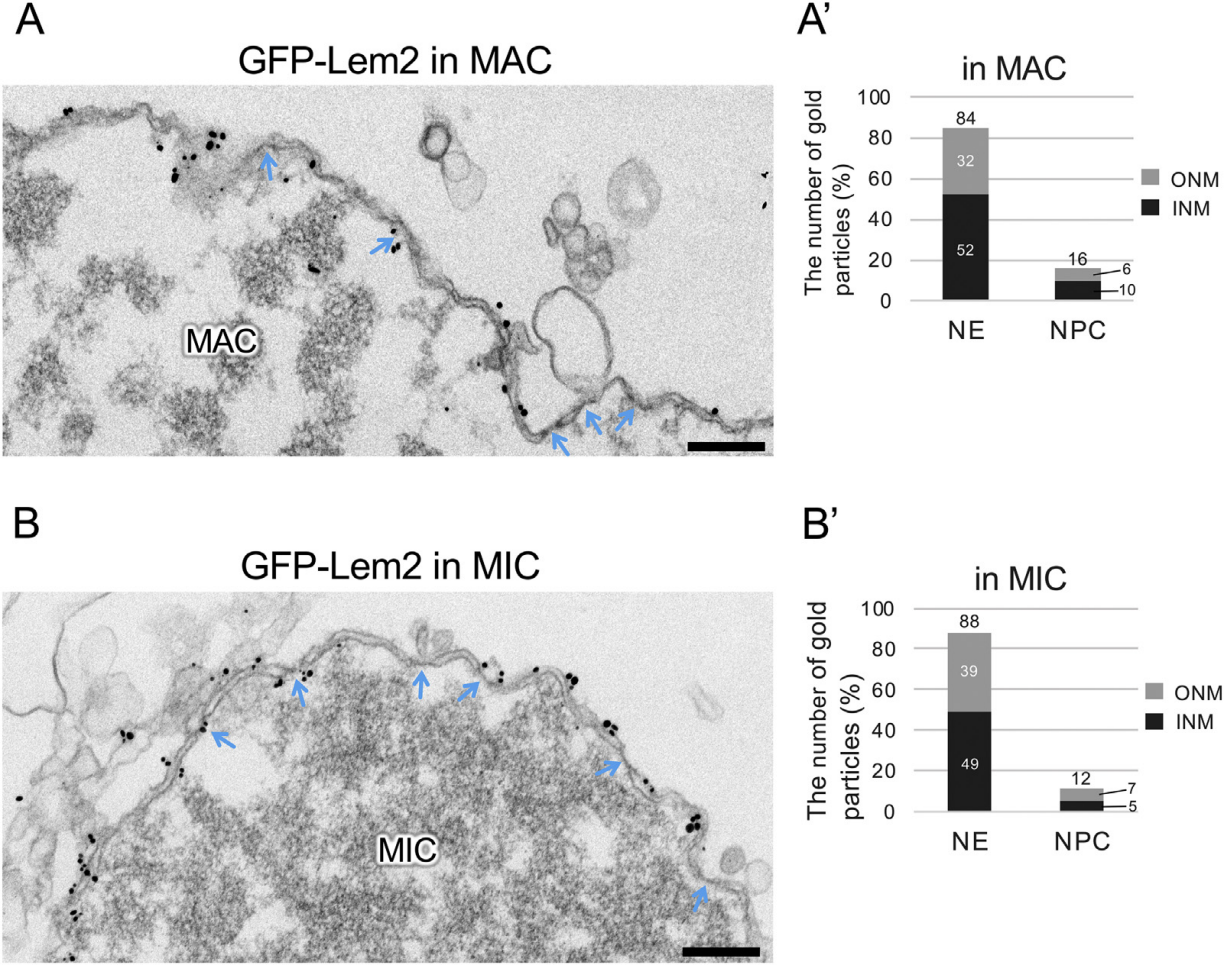
Immunoelectron micrographs for GFP-tagged Lem2 proteins. A. GFP-Lem2 in the MAC. The blue arrows indicate the positions of nuclear pores. The scale bars represent 200 nm. A′. Percentages of the number of the gold particles in the NE and NPC. Darker and brighter bars represent particles located in the nuclear and cytoplasmic sides, respectively. A total of 858 gold particles were counted. B. GFP-Lem2 in the MIC. The blue arrows indicate the positions of nuclear pores. The scale bars represent 200 nm. B′. Percentages of the number of gold particles in the NE and the NPC. Darker and brighter bars represent the particles on the nuclear and cytoplasmic sides, respectively. n = 649. (For interpretation of the references to color in this figure legend, the reader is referred to the web version of this article.)

To further determine the precise localization of these two Lem2 proteins in *Tetrahymena* within the NE, we performed immunoelectron microscopy to detect GFP-fused proteins using anti-GFP antibodies. The gold particles on GFP-Lem2 were localized on both the INM and ONM in the MAC (Fig. 3A); 52% and 32% of particles were on the INM and ONM of the MAC NE, respectively, while the remaining particles (16%) were localized at the MAC NPC (total particle number (n), n = 858; Fig. 3A′). Similarly, particles of GFP-Lem2 were also localized on both the INM and ONM in the MIC (Fig. 3B); 49% and 39% of particles were on the INM and OMN of the MIC NE, respectively, while the remaining particles (12%) were localized at the MIC NPC (n = 649, Fig. 3B′). The localization profile of *Tetrahymena* Lem2 proteins differed from those of other Lem2 proteins in organisms from the Opisthokonta supergroup, which are localized only on the INM (Brachner et al., 2005; Tange et al., 2016). This difference in localization suggests that Lem2 in *Tetrahymena* has a unique feature or unique interacting partner proteins, which determine its localization. Alternatively, this difference may be because of overexpression of the Lem2 protein, as GFP-Lem2 is expressed in the presence of the endogenous untagged protein, or because of GFP-tagging.

**Fig. 4:**
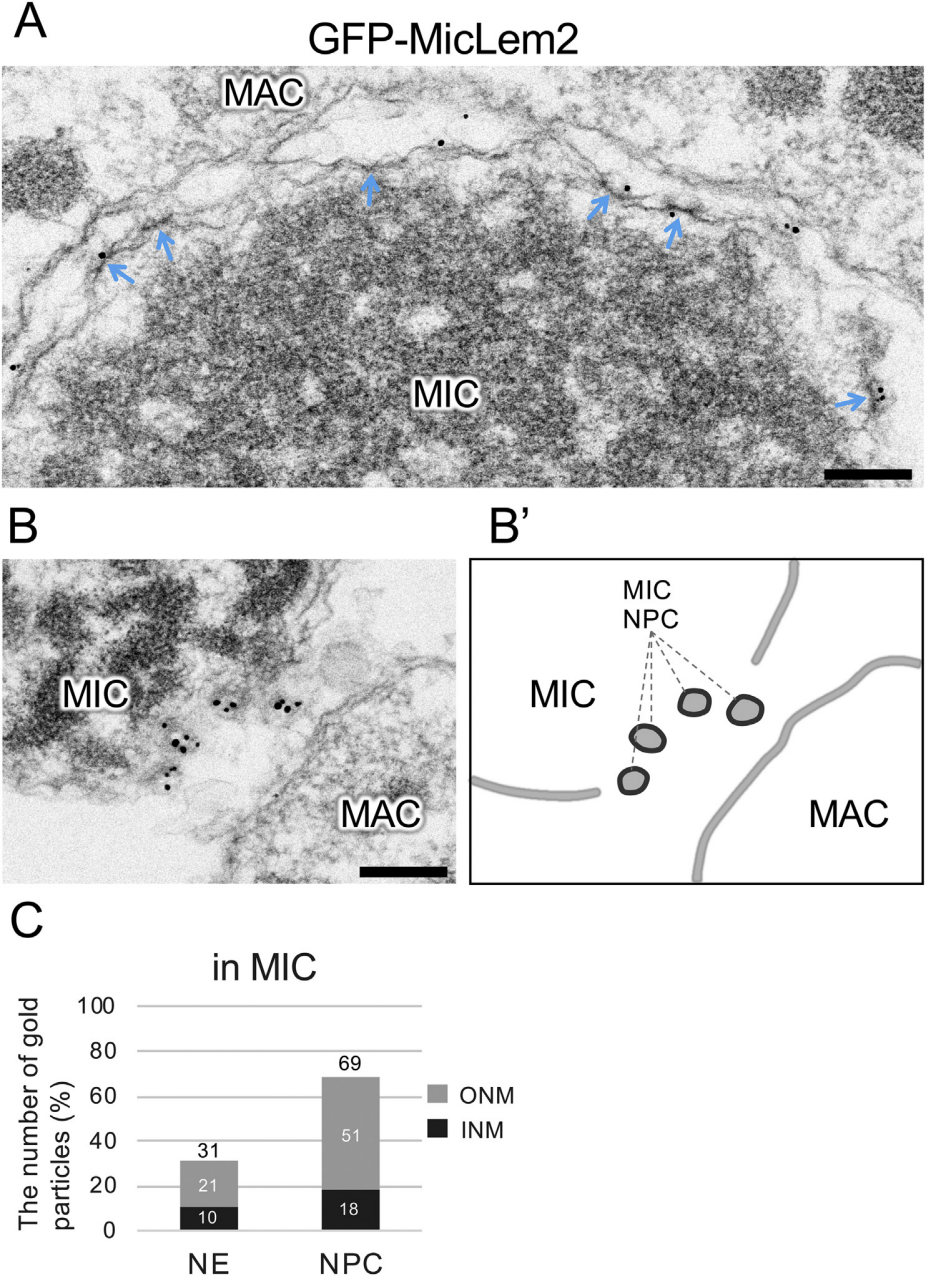
Immunoelectron micrographs for GFP-tagged MicLem2 proteins. “MAC” and “MIC” represent macro- and micronucleus, respectively. The blue arrows indicate the nuclear pores. The scale bars represent 200 nm. A. GFP-MicLem2 in the MIC. B. GFP-MicLem2 in the NPCs of the MIC. B′. Schematic representation of image (B). C. Percentages of the number of the gold particles in the NE and NPC of the MIC. Darker and brighter bars represent particles located on the nuclear and cytoplasmic sides, respectively (n = 464). (For interpretation of the references to color in this figure legend, the reader is referred to the web version of this article.)

On the other hand, gold particles on GFP-MicLem2 were localized on the INM and ONM of the MIC (Fig. 4A). Strikingly, gold particle signals were enriched in the nuclear pore of the MIC (Fig. 4B, B′); 69% and 31% of the particles were on the MIC NPC and the MIC NE, respectively (n = 464, Fig. 4C). The signals on the MIC NPC were enriched on the cytoplasmic side (51%) compared to on the nuclear side (18%) (n = 464, Fig. 4C). Similarly, the signals on the MIC NE were enriched in the ONM (21%) compared to in the INM (10%) (n = 464, Fig. 4C); no significant enrichment of signals was observed on the heterochromatin compared those on the euchromatin. This result explains the punctate localization of GFP-MicLem2 by fluorescence microscopy as shown in Fig. 2E and suggests that MicLem2 associates with MIC-specific nuclear pore complex proteins such as MicNup98A, MicNup98B, MicNup153, MicNup214, and Pom82, as reported previously (Iwamoto et al., 2009; Iwamoto et al., 2017; Iwamoto et al., 2018). Notably, this result may have been affected by overexpression of the MicLem2 protein, as GFP-MicLem2 is expressed in the presence of the endogenous untagged protein, or because of GFP-tagging.

### 3.3 N-terminal domains are responsible for NE localization of the proteins

**Fig. 5:**
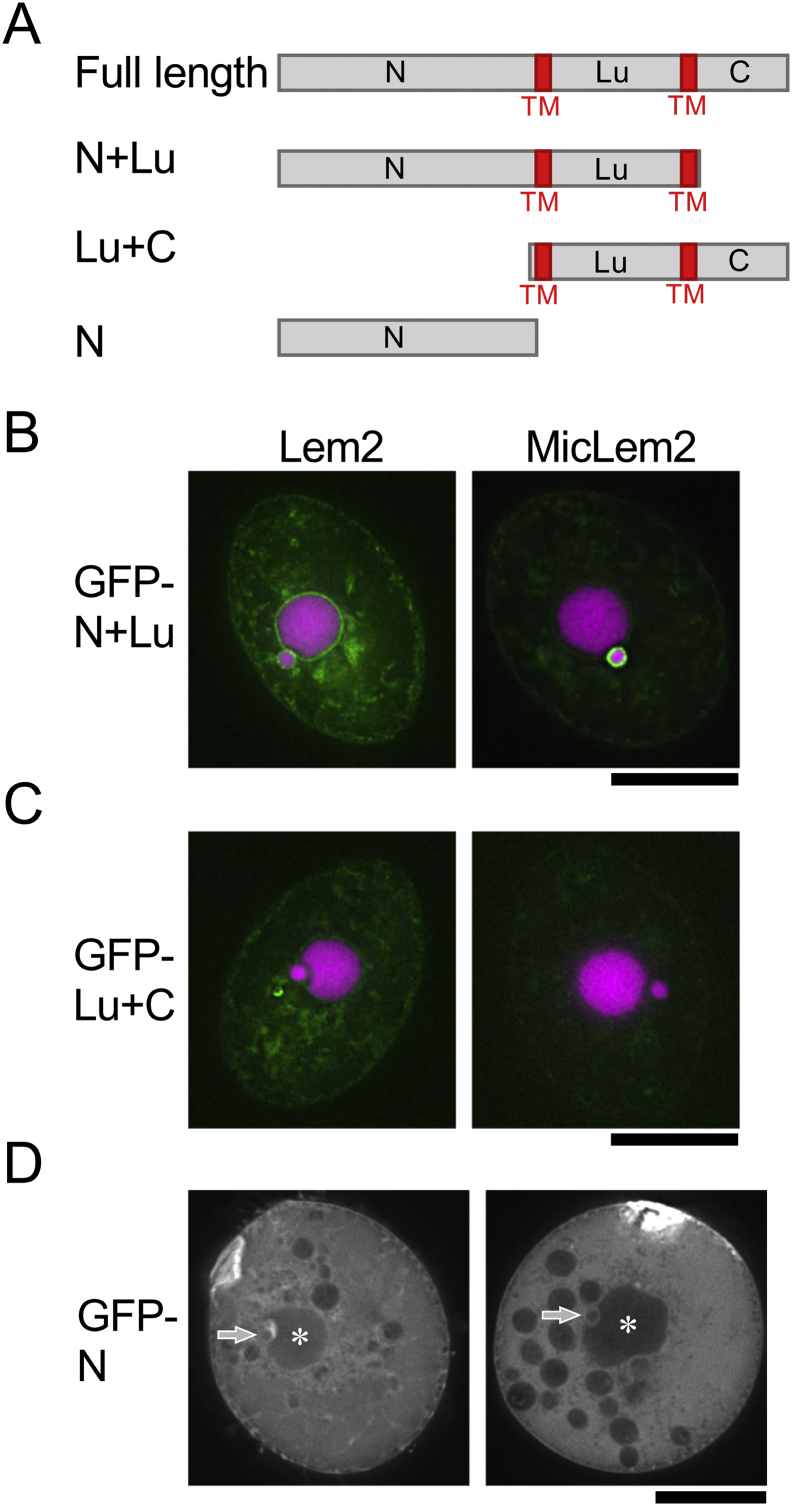
Search for molecular domains responsible for the nuclear envelope localization of *T. thermophila* Lem2 proteins. A. Schematic representation of the tested fragments of Lem2 proteins. “N + Lu” consists of the N-terminal region (N) and luminal region (Lu) with two transmembrane (TM) segments. “Lu + C” consists of Lu and C-terminal region (C) with two TM segments. “N” consists of N without TM segments. B–D. Subcellular localization of GFP-tagged fragments described in (A). B. Subcellular localization of N + Lu fragments of Lem2 and MicLem2. C. Subcellular localization of Lu + C fragments of Lem2 and MicLem2. Cells expressing GFP-tagged fragments were fixed and counterstained with DAPI. The green and magenta colors represent the fluorescence of GFP and DAPI, respectively. D. Subcellular localization of N fragments of Lem2 and MicLem2 in living cells. The asterisks and arrows indicate the MAC and MIC, respectively. The scale bars represent 20 μm. (For interpretation of the references to color in this figure legend, the reader is referred to the web version of this article.)

To understand the domains responsible for NE localization of the proteins, we determined the molecular domains required for NE localization using GFP fusion fragments of the proteins. The names of the molecular domains are shown in Fig. 5A (see details in the legend). For Lem2, the “N + Lu” fragment was predominantly localized in the NE of both the MAC and MIC, similar to the full-length Lem2 (compare left panel of Fig. 5B to Fig. 2A), while the “Lu + C” fragment was not (left panel of Fig. 5C), suggesting that the N-terminal domains but not the C-terminal domains are required for NE localization. MicLem2 was also tested. Similar to Lem2, the “N + Lu” fragment of MicLem2 was predominantly localized in the NE of the MIC, as observed for full-length MicLem2 (compare right panel of Fig. 5B to Fig. 2E), while the “Lu + C” fragment was not (right panel of Fig. 5C), suggesting that the N-terminal domains but not the C-terminal domains are required for localization in the NE of the MIC.

The molecular domains of Lem2 family proteins, which are required for NE localization, have been assigned in other organisms. In mammalian cells, the N-terminal domain with the first transmembrane segment of Man1 is responsible for targeting of the protein to the NE by the diffusion-retention mechanism (Wu et al., 2002). In the fission yeast *S. pombe*, the N-terminal domain of Lem2 is responsible for NE localization by binding to Bqt4, an inner nuclear envelope protein in *S. pombe* (Hirano et al., 2018). In contrast, in the budding yeast *S. cerevisiae*, nuclear localization signals (NLSs) in the N-terminal regions of Heh2 are responsible for NE localization (King et al., 2006; Lokareddy et al., 2015). We examined whether the N-terminal domains of Lem2 and MicLem2 in *Tetrahymena* contain NLSs, similar to the case in *S. cerevisiae*. We observed the localization of GFP-fused N-terminal fragments (N), which lack transmembrane helix segments. Fluorescence signals of GFP-Lem2-N and GFP-MicLem2-N showed diffused localization in the cytoplasm, but neither localized in either the MAC or MIC (Fig. 5D). This suggests that Lem2 and MicLem2 of *Tetrahymena* do not contain NLS sequences in their N-terminal domains, unlike *S. cerevisiae*. Because the N + Lu fragments of Lem2 are localized in the NEs of the MAC and MIC, the N-terminal domain with the transmembrane segment plays a role in retaining the proteins in the NE, as observed in mammalian cells and *S. pombe*. Additionally, the N + Lu fragment of the MicLem2 is specifically localized to the NE of the MIC but not to that of the MAC; this fragment appears to bind some proteins specific to the MIC. If this occurs, the NE localization of Lem2 and MicLem2 in *Tetrahymena* may be driven by their tethering to some NE proteins or NPC proteins. This hypothesis is supported by the observation that Lem2 in *S. pombe* localizes in the NE by binding to Bqt4.

### 3.4 Behaviors of Lem2 proteins during sexual reproduction

**Fig. 6:**
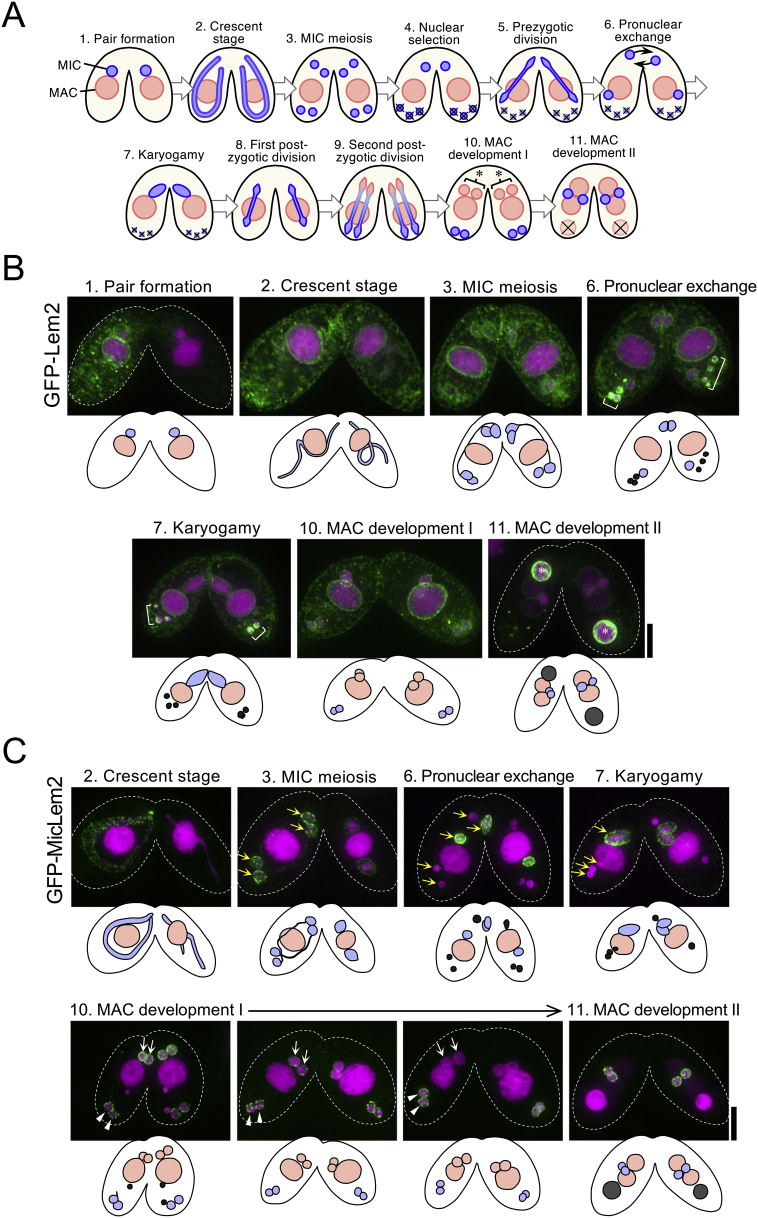
Subcellular localization of Lem2 proteins during conjugation of *T. thermophila*. A. Schematic representation of typical nuclear events occurring during conjugation. Classification of the stages and typical appearance of the cell are shown in the drawings. Nuclei exhibiting the characteristics of the MIC are shown in blue and those exhibiting the characteristics of the MAC are shown in pink. The asterisks indicate presumptive new macronuclei located in the anterior cytoplasm at the stage of “MAC development I”. The X-marks indicate the nuclei being degraded. B. Fluorescence images of GFP-Lem2 (upper panels) and their drawings (lower drawings) in conjugating pairs. The pairs were fixed and stained with DAPI. The green and magenta colors represent the fluorescence of GFP and DAPI, respectively. The white broken line outlines a conjugating pair. The brackets indicate the unselected haploid nuclei being degraded. C. Fluorescence images of GFP-MicLem2 (upper panels) and their drawings (lower drawings) in conjugating pairs. The pairs were fixed and stained with DAPI. The yellow arrows indicate the MIC-derived nuclei at the stages of “MIC meiosis”, “Pronuclear exchange”, and “Karyogamy.” White arrows and arrowheads indicate nuclei differentiating to new macronuclei and new micronuclei, respectively, during the “MAC development I” stage. The scale bars represent 10 μm. (For interpretation of the references to color in this figure legend, the reader is referred to the web version of this article.)

Because the expression of Lem2 and MicLem2 is upregulated during conjugation, a sexual reproduction process in ciliates (expression profiles are available at http://ciliate.org/index.php/feature/details//TTHERM_00540280 for Lem2 and TTHERM_00145310 for MicLem2 in the *Tetrahymena* Genome Database; also see Supplemental Fig. S2), these two Lem2 proteins may play stage-specific roles in conjugation. To understand the roles of these proteins in ciliates, we examined the dynamic behaviors of these proteins during conjugation using GFP-fusion proteins (schematically represented in Fig. 6A).

For conjugation of a mating pair of cells, a *Tetrahymena* strain expressing GFP-Lem2 was mated with the wild-type strain not expressing GFP-fusion proteins. After conjugation, the GFP-fusion proteins moved to the mating partner cell to stain the targeted organelle, as described previously (Iwamoto et al., 2015). Upon conjugation, GFP-Lem2 was mainly localized in the NE of the MAC and MIC and partially in various membranes in the cytoplasm in cells expressing the protein in the initial stage of conjugation (Pair formation); next, GFP-Lem2 fluorescently stained the NE of the MAC and MIC and other membranes of the partner cell throughout the conjugation process, from the “Crescent” to “MAC development II” stages. Additionally, two types of degenerating nuclei were fluorescently stained: one was unselected haploid nuclei typically located in the posterior cytoplasm often appearing in the panels “Pronuclear exchange” and “Karyogamy” of Fig. 6B (see nuclei marked by single parentheses), while the other one is a parental MAC (marked by asterisks in Fig. 6B), which appeared in the “MAC development II” stage (Fig. 6B). This suggests that Lem2 in *Tetrahymena* plays a role in marking the nuclei to be degraded; however, whether it acts as an “eat-me” signal remains unclear.

In contrast, GFP-MicLem2 was strictly localized to the NEs of the MIC and MIC-derived nuclei throughout the conjugation process (Fig. 6C). A punctate distribution of GFP-MicLem2, similar to the case in vegetative growing cells (Fig. 2E), was observed in the nuclei of the “Crescent” (meiotic prophase) and “MIC meiosis” stages (Fig. 6C). However, in later stages, such as “Pronuclear exchange” and “Karyogamy”, fluorescence signals of GFP-MicLem2 were lost from the unselected haploid nuclei to be degraded (compare yellow arrows in panels of “MIC meiosis”, “Pronuclear exchange”, and “Karyogamy” in Fig. 6C). Interestingly, during the early stage of macronuclear development (“MAC development I”), the fluorescence signal of GFP-MicLem2 was promptly removed from the presumptive new macronuclei localized in the anterior cytoplasm (compare white arrows in the left-most panel of the “MAC development I” stage with those in the right two panels in Fig. 6C), whereas the signal remained in the presumptive new micronuclei localized in the posterior cytoplasm (see arrowheads in Fig. 6C). These results suggest that MicLem2 plays a role in marking MICs to prevent their degradation.

## 4 Discussion

One of the characteristic features of LEM domain proteins is the presence of an LEM domain, which is a bi-helical motif, in the N-terminus (Laguri et al., 2001; Cai et al., 2001). However, the amino acid sequence of this motif is not well-conserved in eukaryotes other than metazoan (Brachner and Foisner, 2011). Instead, the predicted HeH/SAP motif is present in Lem2-related proteins in *S. cerevisiae* and *S. pombe* (Fig. 1A). Although the presence of some Lem2-related proteins has been suggested in various eukaryotes including *Arabidopsis*, *Blastocystis*, *Trichomonas*, *Dictyostelium*, and *Tetrahymena* (Brachner and Foisner, 2011), these motifs were not conserved, and therefore Lem2-related proteins were not identified in these eukaryotes (Mans et al., 2004). In this study, we used the MSC domain as a query because its amino acids sequence was well-conserved among Lem2-related proteins from various eukaryotes including *Tetrahymena* (Supplemental Fig. S1B) and we identified two Lem2 homolog proteins from *T. thermophila*. Because *Tetrahymena*, belonging to the SAR supergroup, is distant from animals and yeasts belonging to the Opisthokonta supergroup (see Fig. 1B), identifying homologs in *Tetrahymena* is important for understanding the evolutional divergence of NE proteins in eukaryotes. Additionally, we identified genes (GAQ87803, XP_015627899, NP_199468) encoding potential homologs of the Lem2-related protein in species belonging to the Archaeplastida supergroup (Fig. 1C). Because plants are also not closely related to animals, fungi, and ciliates, identification of the plant homolog, together with *Tetrahymena* homologs, contributes to the understanding of evolutional divergence of NE protein.

*Tetrahymena* Lem2 is localized in both ONM and INM of the NE (Fig. 2). This localization pattern contrasts that of Lem2 in mammalian (Brachner et al., 2005) and fission yeast cells (Tange et al., 2016), in which this protein is localized only in the INM of the NE. A possible explanation for this difference is the effect of protein overexpression. However, this is unlikely because spontaneously increased or decreased expression of Lem2 in *Tetrahymena* cells does not alter its localization. MicLem2 is enriched in the MIC NPCs (Fig. 2). This striking feature in localization has not been observed previously for Lem2 homologs in other organisms. The factors that determine and regulate the localization of Lem2 homolog requires further analysis. In *S. pombe*, a portion within 100 amino acids of the N-terminal region immediately before the first TM domain binds Bqt4, another INM protein, and its binding to Bqt4 determines its NE localization (Hirano et al., 2018); recently, Bqt4-binding domain of Lem2 was further narrowed to a single α-helix of 19 amino acids (Hu et al., 2018). This clearly demonstrates that Lem2 localization is regulated by other protein factors. If this is the case, *Tetrahymena* Lem2 interacts with cytoplasmic proteins and nuclear proteins including nucleoporins, and MicLem2 interacts with MIC-NPC-specific nucleoporins. The nucleoporins composing of the MAC- and MIC-NPCs are known (Iwamoto et al., 2009; Iwamoto et al., 2017; Iwamoto et al., 2018); Pom82, MicNup214, MicNup98A, MicNup98B, and MicNup153 are specific to MIC-NPC, while Pom121, MacNup214, MacNup98A, MacNup98B, and MacNup153 are specific to MAC-NPC (Malone et al., 2008; Iwamoto et al., 2009; Iwamoto et al., 2017; Iwamoto et al., 2018). These MIC-NPC-specific nucleoporins may act as key players in anchoring MicLem2 to the MIC-NPC. Additionally, the presence of numerous α-helices in the N-terminal region of MicLem2 compared to in other Lem2 proteins may be important for its MIC NPC localization (Fig. 1A). This is because these α-helices may form a platform structure for interacting with MIC-NPC-specific nucleoporins. In fact, a single α-helix of *S. pombe* Lem2 is sufficient to bind Bqt4, which anchors Lem2 to the NE as described above, supporting this idea.

The unique yet characteristic localization patterns of *Tetrahymena* Lem2 and MicLem2 may reflect their functions. Dynamic changes in their localization during conjugation appear to be correlated with their functions in nuclear degradation; Lem2 is enriched in the nuclei where it is later degraded, whereas MicLem2 is enriched in the nucleus to ensure cell survival (Fig. 6). It has been reported that some nuclei, which are specifically selected by unknown mechanisms, are broken down by a specific type of autophagy (known as nucleophagy) during conjugation (Akematsu et al., 2010; Liu and Yao, 2012; reviewed in Mijaljica and Devenish, 2013). It has been reported that the nucleus in cells lacking emerin, one of the LEM domain proteins in humans, is frequently degraded by nucleophagy (Park et al., 2009). The presence or absence of Lem2 or MicLem2 may determine nucleus survival or degradation. Our results indicate that Lem2 and MicLem2 are good markers of selected (to survive) and unselected (to be degraded) nuclei, respectively, during conjugation.

In this study, two Lem2 family proteins, Lem2 and MicLem2, were identified in *T. thermophila*, which belongs to the Alveolata group in the SAR supergroup. This indicates that Lem2 is evolutionarily highly conserved in eukaryotes. Our findings in *Tetrahymena* improve the understanding of NE functions which are evolutionarily conserved among a wide variety of eukaryotes and those that diverged between species during evolution.

The following are the supplementary data related to this article.

Supplemental Table 1 Primers used in this study. Supplemental Table 1

Supplementary Fig. S1 Multiple sequence alignment of the LEM/HeH and the MSC domains. *Homo sapiens* (*Hs*) LEM2, *Hs* MAN1, *Caenorhabditis elegans* Lem2, *Schizosaccharomyces pombe* (*Sp*) Lem2, *Sp* Man1, *Saccharomyces cerevisiae* (*Sc*) Heh2p, *Sc* Src1p, *Tetrahymena thermophila* (*Tt*) Lem2, and *Tt* MicLem2 were aligned by ClustalX with all parameters set to default. A. Alignment of the LEM/HeH domain. B. Alignment of the MSC domain. Supplementary Fig. S1

Supplementary Fig. S2 Expression profiles of the Tetrahymena proteins possessing the MSC domain. The data was retrieved from TetraFGD (http://tfgd.ihb.ac.cn/). The averaged value of two independent experiments presented in the database are plotted. The horizontal axis represents successive stages of the cultured cells which mRNA was extracted from. Ll, Lm, and Lh represent low, medium, and high cell concentrations in logarithmic growth condition, respectively. For starvation and conjugation stages, numbers represent hours after the transfer of the cells to starvation and conjugation conditions, respectively. The vertical axis represents the values of mRNA expression. More details are available in the database website. Supplementary Fig. S2

Supplemental data 1 Aligned amino acid sequences of full length Lem2-related proteins. Supplemental data 1

Supplemental data 2 Amino acid sequences of the MSC domain of Lem2-related proteins to generate the phylogenic tree in Fig. 1C. Supplemental data 2

Supplementary material Image 1

## Competing interests

The authors declare no competing financial interests.

## Author contributions

MI and TH designed the research. MI, YF, HO, and CM performed experiments. MI, YF, YH, and TH analyzed the data and wrote the manuscript. All authors read and approved the final manuscript.
