# Supplementary material for: Identification of the evolutionarily conserved nuclear envelope proteins Lem2 and MicLem2 in Tetrahymena thermophila
Source: Gene X. 2019 Jan 22;1:100006. doi: 10.1016/j.gene.2019.100006 (PMC7285967; doi:10.1016/j.gene.2019.100006)
Supplement: Supplemental Table 1 — Primers used in this study. [file mmc1.docx]

**Supplemental Table 1**. Primers used in this study.

| Target | Primer name | Sequence (5’ → 3’) |
| --- | --- | --- |
| TTHERM_00540280 (Lem2)  Full length for N-term tag | 00540280/F(XhoI)  00540280/R(ApaI) | GGCTCGAGATGAGCAAACCTGTCAAT  CCGGGCCCTCAAATTTAAGACCAATATAGTCC |
| Full length for C-term tag | 00540280/F(XhoI)  00540280/R2(KpnI) | GGCTCGAGATGAGCAAACCTGTCAAT  ATGGTACCTTAAATTTAAGACCAATATAGTCC |
| N+Lu fragment | 00540280/F(XhoI)  00540280/R3(ApaI) | GGCTCGAGATGAGCAAACCTGTCAAT  ATGGGCCCTCAGTGCTTCTTTAAAATTATTCCAAC |
| Lu+C fragment | 00540280/F2(XhoI)  00540280/R(ApaI) | ATCTCGAGATGAAAATCAATTATTCACTTTACTTC  CCGGGCCCTCAAATTTAAGACCAATATAGTCC |
| N fragment | 00540280/F(XhoI)  00540280dTM/R(ApaI) | GGCTCGAGATGAGCAAACCTGTCAAT  TAGGGCCCTCAGATTTTAGATGGATTATAAG |
| TTHERM_00145310 (MicLem2) |  |  |
| Full length for N-term tag | 00145310/F(XhoI)  00145310/R(ApaI) | GGCTCGAGATGAATTCAGATCAATAAAATAGG  GCGGGCCCTCAACTTAGCTTTCCCCTTCTTTG |
| Full length for C-term tag | 00145310/F(XhoI)  00145310/R2(KpnI) | GGCTCGAGATGAATTCAGATCAATAAAATAGG  ATGGTACCTTAACTTAGCTTTCCCCTTCTTTG |
| N+Lu fragment | 00145310/F(XhoI)  00145310/R3(ApaI) | GGCTCGAGATGAATTCAGATCAATAAAATAGG  ATGGGCCCTCAAAATCTCCTAACTGCTGCCAC |
| Lu+C fragment | 00145310/F2(XhoI)  00145310/R(ApaI) | ATCTCGAGATGTTCTCAAAAATAGAAATTAGC  GCGGGCCCTCAACTTAGCTTTCCCCTTCTTTG |
| N fragment | 00145310/F(XhoI)  00145310dTM/R(ApaI) | GGCTCGAGATGAATTCAGATCAATAAAATAGG  TAGGGCCCTCATGAGAAAAATATCAAGAC |
