# Supplementary material for: Identification of the evolutionarily conserved nuclear envelope proteins Lem2 and MicLem2 in Tetrahymena thermophila
Source: Gene X. 2019 Jan 22;1:100006. doi: 10.1016/j.gene.2019.100006 (PMC7285967; doi:10.1016/j.gene.2019.100006)
Supplement: Supplementary Fig. S1 — Multiple sequence alignment of the LEM/HeH and the MSC domains. Homo sapiens (Hs) LEM2, Hs MAN1, Caenorhabditis elegans Lem2, Schizosaccharomyces pombe (Sp) Lem2, Sp Man1, Saccharomyces cerevisiae (Sc) Heh2p, Sc Src1p, Tetrahymena thermophila (Tt) Lem2, and Tt MicLem2 were aligned by ClustalX with all parameters set to default. A. Alignment of the LEM/HeH domain. B. Alignment of the MSC domain. [file mmc2.pdf]

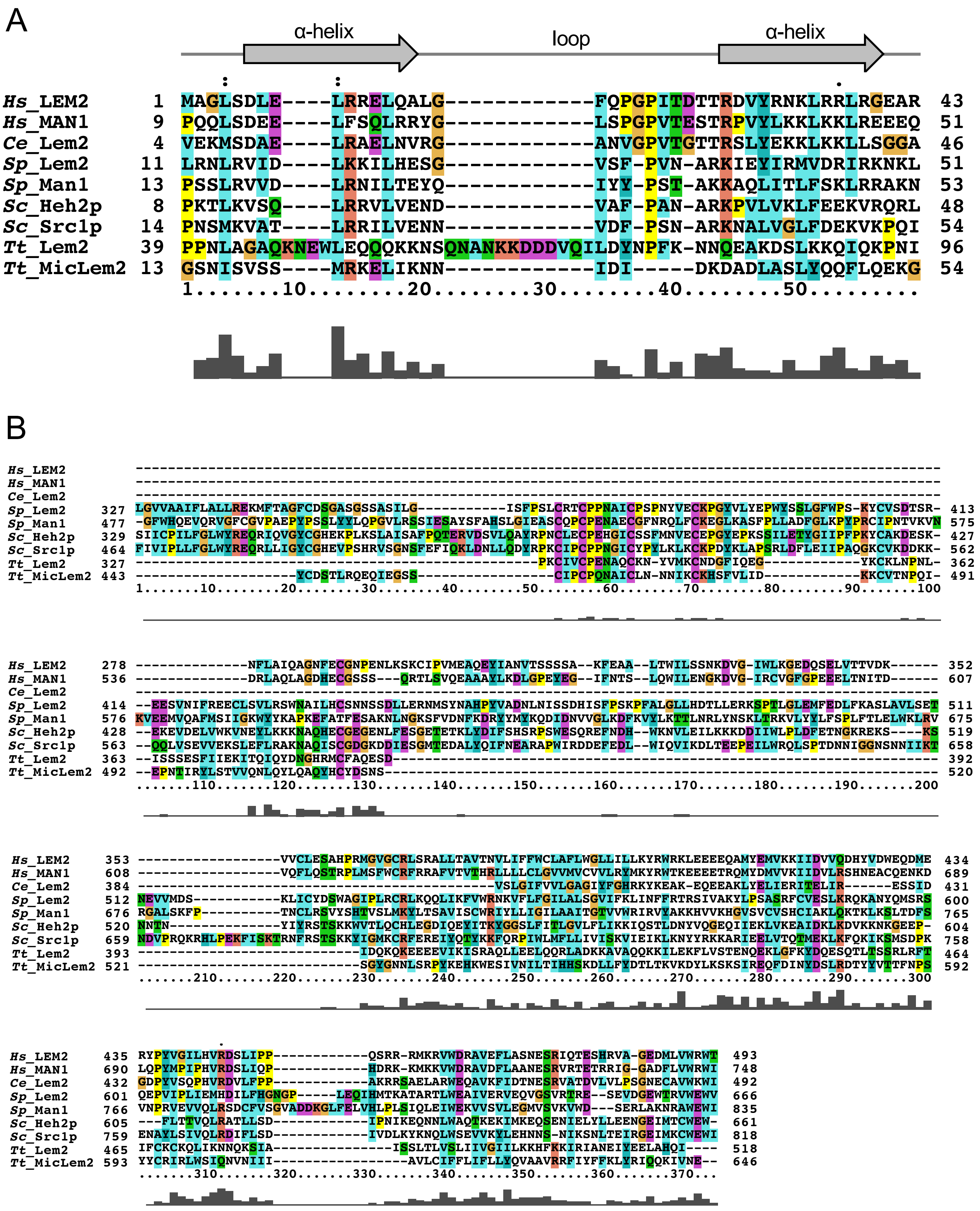

Supplementary Figure S1. Multiple sequence alignment of the LEM/HeH and the MSC domains. *Homo sapiens (Hs)* LEM2, *Hs* MAN1, *Caenorhabditis elegans* Lem2, *Schizosaccharomyces pombe (Sp)* Lem2, *Sp* Man1, *Saccharomyces cerevisiae (Sc)* Heh2p, *Sc* Src1p, *Tetrahymena thermophila (Tt)* Lem2, and *Tt* MicLem2 were aligned by ClustalX with all parameters set to default. A. Alignment of the LEM/HeH domain. B. Alignment of the MSC domain.
