# Supplementary material for: Identification of the evolutionarily conserved nuclear envelope proteins Lem2 and MicLem2 in Tetrahymena thermophila
Source: Gene X. 2019 Jan 22;1:100006. doi: 10.1016/j.gene.2019.100006 (PMC7285967; doi:10.1016/j.gene.2019.100006)
Supplement: Supplementary Fig. S2 — Expression profiles of the Tetrahymena proteins possessing the MSC domain. The data was retrieved from TetraFGD (http://tfgd.ihb.ac.cn/). The averaged value of two independent experiments presented in the database are plotted. The horizontal axis represents successive stages of the cultured cells which mRNA was extracted from. Ll, Lm, and Lh represent low, medium, and high cell concentrations in logarithmic growth condition, respectively. For starvation and conjugation stages, numbers represent hours after the transfer of the cells to starvation and conjugation conditions, respectively. The vertical axis represents the values of mRNA expression. More details are available in the database website. [file mmc3.pdf]

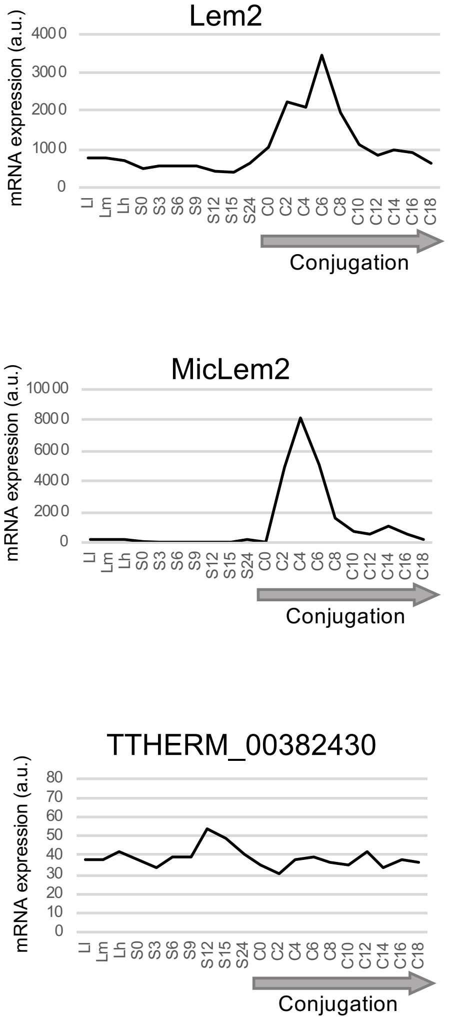

Supplementary Figure S2. Expression profiles of the Tetrahymena proteins possessing the MSC domain. The data was retrieved from TetraFGD (http://tfgd.ihb.ac.cn/). The averaged value of two independent experiments presented in the database are plotted. The horizontal axis represents successive stages of the cultured cells which mRNA was extracted from. Ll, Lm, and Lh represent low, medium, and high cell concentrations in logarithmic growth condition, respectively. For starvation and conjugation stages, numbers represent hours after the transfer of the cells to starvation and conjugation conditions, respectively. The vertical axis represents the values of mRNA expression. More details are available in the database website.
